# Fat Intake Reduction Strategies among Children and Adults to Eliminate Obesity and Non-Communicable Diseases in the Eastern Mediterranean Region

**DOI:** 10.3390/children5070089

**Published:** 2018-06-29

**Authors:** Ayoub Al Jawaldeh, Hanin Al-Jawaldeh

**Affiliations:** 1 Department of Nutrition Sciences, University of Vienna, UZA2 Althantstrasse 14, 1090 Vienna, Austria; 2 Health Science Department, The American University of Madaba, Madaba 11821, Jordan; haljawaldeh@yahoo.ca

**Keywords:** NCD, Eastern Mediterranean region, fat, SFAs, TFAs

## Abstract

Non-communicable diseases (NCDs) are the leading cause of mortality globally with an estimated 39.5 million deaths per year (72% of total death) in 2016, due to the four major NCDs: cardiovascular diseases, cancers, chronic respiratory diseases and diabetes. In the Eastern Mediterranean Region (EMR), almost 68% of all deaths are attributed to NCDs commonly known as chronic or lifestyle-related diseases. Two-thirds of NCD premature deaths are linked to 4 shared modifiable behavioral risk factors: tobacco use, unhealthy diet, physical inactivity and harmful use of alcohol. These unhealthy behaviours lead to 4 key metabolic/biological changes; raised blood pressure, overweight/obesity, high blood glucose levels/diabetes, and hyperlipidemia (high levels of fat in the blood), that increase the risk of NCDs. Globally, countries are already working towards agreed global goals on maternal and infant nutrition and on the prevention of NCDs. In both fields the goals include halting the increase in overweight and obesity and reducing NCD diet-related risk factors including reducing saturated fatty acids (SFAs) and trans fatty acids (TFAs) intake. The objective of this review is to present an up-to-date overview of the current fat (SFAs and TFAs) intake reduction initiatives in countries of the Eastern Mediterranean Region (EMR) by highlighting national and regional programs, strategies and activities aiming at decreasing the intakes of dietary fat (SFA and TFA). The literature review shows that the average intake of SFA is estimated to be 10.3% of the total energy intake (EI), exceeding the WHO (World Health Organization) upper limit of 10%. The average TFA intake is estimated at 1.9% EI, which also exceeds the WHO upper limit of 1% EI. The highest SFAs intake was reported from Djibouti, Kuwait, Saudi-Arabia, Lebanon and Yemen, while the highest TFAs intakes were reported from Egypt and Pakistan. If countries of the EMR receive immediate public health attention, that toll of NCD-related morbidity and mortality would be considerably decreased through the implantation of evidence-based preventive interventions. In this context, reductions in SFAs and TFAs intakes have been highlighted as cost-effectives strategies that may hamper the growth of the NCD epidemic.

## 1. Introduction

The global burden of Non-Communicable Diseases (NCDs) represents a major public health challenge throughout the world [1,2]. NCDs are the leading cause of mortality globally, representing 72% of total deaths [3]. NCDs are also responsible for 16 million premature deaths (before the age of 70), killing people in a productive age [1]. Currently, around 68% of deaths in the Eastern Mediterranean Region (EMR) are attributed to NCDs [1]. The WHO (World Health Organization) EMR is one of the six official WHO-designated geographical areas, comprising 22 countries and territories (Afghanistan, Bahrain, Djibouti, Egypt, Iran, Iraq, Jordan, Kuwait, Lebanon, Libya, Morocco, Oman, Pakistan, Palestine, Qatar, Saudi Arabia, Somalia, Sudan, Syria, Tunisia, United Arab Emirates (UAE) and Yemen), with a total estimated population of 550 million [4]. There is substantial variation in terms of population health outcomes, health care infrastructure and quality and level of health expenditure. This variability is largely related to the economic development in a region that consists of low-, middle- and high-income countries [4].

The regional obesity and overweight prevalence rates are above global average with about 50% of adult women and 43.8% of adult men being overweight or obese in 2014 [5]. In several countries, more than 65% of adults (especially women) are overweight or obese. Furthermore, high rates of childhood (6.9% of children under five years) are already overweight—higher than the global average of 6.2%—and in some countries more than 15% of children are overweight or obese. In many countries of the region, more than 50% of adolescents are overweight or obese [5]. Moreover, around 43 million people in the region live with diabetes, affecting more than 20% of adults in some countries. The Region has the highest death rates from diabetes of all WHO regions with more than 10% of deaths in men and 9% of deaths in women attributed to diabetes [5]. The total deaths caused by cardiovascular diseases (CVDs) in EMR is 1.3 million in 2016, representing 31.9% of total deaths (Table 1) [6].

**Figure 1:**
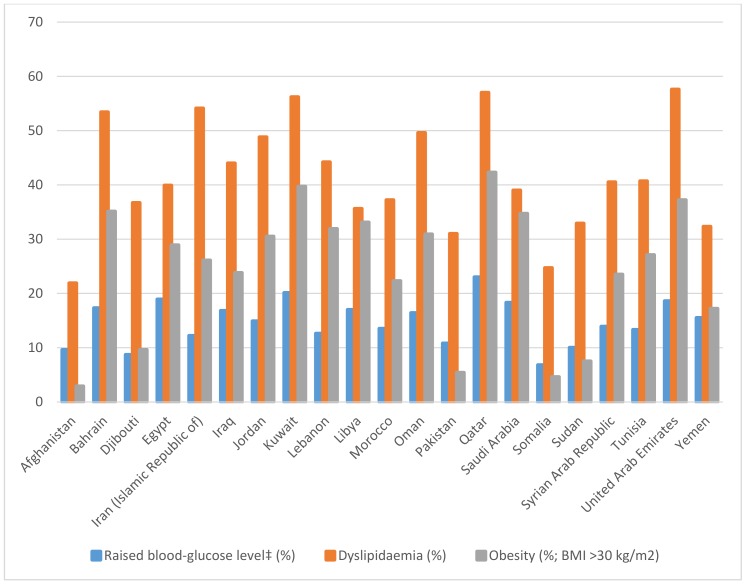
Prevalence of obesity, dyslipidaemia and impaired glucose levels in EMR (Eastern Mediterranean Region). BMI = body mass index.

The global prevalence of elevated total cholesterol (≥5 mmol/L) among adults aged ≥25 years was 38.9% (37.3% for men and 40.2% for women) [7]. Among the WHO-designated regions, the prevalence of hypercholesterolaemia was the third highest in the world, at 38.4% (40.4% for women and 36.2% for men) [7]. The prevalence of hypercholesterolaemia was ≥50% in the majority of Gulf Countries, with the highest prevalence in UAE at 56.7% (Figure 1). The trend of obesity, dyslipidaemia and raised blood-glucose levels are going on the same trend in all countries of the Region, which reflect the association of these risks factors with nutrition transition in the Region, especially in middle- and high-income countries [1,5,7].

### Impacts of Reducing SFAs and TFAs Intake among Children and Adults

Fat consists of Trans Fatty Acids (TFAs), Saturated Fatty Acids (SFAs) and unsaturated fatty acids (UFAs). SFAs are fatty acids consisting solely of single carbon–carbon bonds (i.e., no double bonds). They are found in foods from animal sources such as butter, cow’s milk, meat, salmon and egg yolks, and some plant-derived products such as chocolate and cocoa butter, coconut, palm and palm kernel oils. UFAs are naturally occurring in foods such as fish, avocado, nuts, sunflower, canola and olive oils. TFAs are unsaturated fats found in foods obtained from ruminants, such as dairy products and meat, and in industrially produced partially hydrogenated vegetable oils. TFAs are typically found in processed food, fast food, snack food, fried food, frozen pizza, pies, cookies, margarines and spreads [8,9]. Even though fat consumption provides the body with energy, supports cell growth, protects body organs and keeps it warm, the excess consumption of SFAs and TFAs is unhealthy. Moreover, consumption of TFAs, especially industrially produced partially hydrogenated vegetable oils, has been associated with an increased risk of heart disease, infertility, endometriosis, gallstones, Alzheimer’s disease, diabetes and some cancers [8,10]. Increased intake of TFAs (>1% of total energy intake) is associated with increased risk of Coronary Heart Disease (CHD) mortality and events [11,12]. Globally, more than 500,000 deaths in 2010 were attributed to the increased intake of TFAs [12].

A major mechanism, by which SFAs increase the risk of CVDs, is due to increased production of low-density lipoprotein cholesterol (LDL-C) in the liver in response to the increased levels of SFA in the diet. This in turn is linked to the disruption of blood lipid profile including elevated total cholesterol (TC) and LDL-C, siding SFAs among the leading risk factors for CVDs such as strokes and heart diseases [13,14]. LDL cholesterol contributes to the narrowing and calcification of arteries, a process known as atherosclerosis. If an artery narrows to the point that the blood flow is impaired, cardiovascular events such as stroke and myocardial infarction may occur. Reducing SFA intake would lower both LDL-C and high-density lipoprotein cholesterol (HDL-C); however, the magnitude of the reduction is greater for LDL-C compared to HDL-C. Micha & Mozaffarian (2010) have evaluated SFA’s effects on cardio metabolic risk factors, CHD, stroke and diabetes, concluding that the TC:HDL-C ratio is significantly decreased by the consumption of lauric acid, when compared to carbohydrate consumption [15]. Evidence shows that increased inflammation with increase of saturated fatty acid intake may also be involved in this process. While the available evidence is strongest for replacing SFAs with Poly-Unsaturated Fatty Acids (PUFAs) first, in terms of lowering LDL-C and reducing the risk of CVDs, followed by Mono-Unsaturated Fatty Acids (MUFAs) and then by carbohydrates, specifically the unrefined type [11,16,17,18]. In fact, when looking at a substitution of 5% of the energy intake between SFAs and PUFAs, by replacing SFAs with PUFAs, the risk of CHD is decreases by 10% [11]. Evidence suggests that replacing SFAs and TFAs with MUFAs such as olive oil may also be a healthy way to reduce SFAs and TFAs intake.

However, it is crucial to note that recent meta-analyses re-evaluated the association between SFAs and CVDs, concluding that there is no significant evidence associating the consumption of SFAs with CHD; therefore, it is imperative to investigate the nutrients replacing SFA when the latter’s consumption is modified [19,20]. As for SFA’s effect on vascular function, diabetes and insulin resistance, further research is needed as evidence in this area is still inconclusive [19]; however, given the suggested biologically possible mechanisms implicating SFA in the etiology of insulin resistance, it is recommended to limit the intake of dietary SFAs [21].

Although CVDs typically appear later in life, lesions in the aorta and coronary arteries that can signal the beginning of artery narrowing or ‘clogging’ (i.e., atherosclerosis) can begin to appear in childhood and are positively associated with dyslipidaemia (i.e., unhealthy changes in blood lipids such as cholesterol and triglycerides) and other CVDs risk factors. Elevated total and LDL-C in childhood are in turn associated with an increase in CVDs risk factors in adulthood, including thickening of the inner layers of the carotid artery which is an indication of subclinical atherosclerosis and predictor of future cardiovascular disease [22].

The quality of available evidence for the effect of reducing SFAs intake was considered to be high in lowering total cholesterol, LDL-C, and diastolic blood pressure, except for systolic blood pressure, triglycerides, waist circumference and insulin resistance, for all of which the quality was considered to be moderate [22,23].

Industrially produced TFAs have no health benefits and should be considered as food additive. Elimination of industrially produced TFAs is feasible and achievable. It is important to note that reducing TFAs lowers LDL-C regardless of whether TFAs were replaced with PUFAs, MUFAs or carbohydrates. TFA consumption has also been shown to reduce the uptake of triglyceride (TG) by the liver [24,25], highlighting the implication of TFAs in the lipid triad which is a documented risk factor for CVDs. In addition to this, other CVDs risk factors have been linked to increased TFAs consumption, including increased platelet aggregation [14], increased plasma inflammatory markers (E-selectin and C-reactive protein) and endothelial function disruption [26,27]. Evidence is also suggesting a role for TFAs in increasing the risk of other metabolic changes central to NCDs, namely obesity, insulin resistance, and metabolic syndrome [25,28,29]. The role of TFA in the etiology of type 2 diabetes has not been as extensively investigated compared to its role in CHD pathogenesis. However, the Nurses’ Health Study has shown a clear dose–response relation between TFAs consumption and type 2 diabetes due to TFA’s inflammatory cascade response [30]. As for obesity, increased consumption of TFAs promotes abdominal fat deposition and eventually weight gain [31,32]. The role of TFAs in early life nutrition and its link with NCDs has been also proposed, with TFAs reported to interfere with fetal essential fatty acid metabolism, thus affecting proper fetal growth and development [33]. Although there have been few studies of trans-fatty acid intake in children, results of dietary intervention studies conducted in children have demonstrated significant reductions in total cholesterol, LDL cholesterol, or both, when saturated fatty acids were replaced with PUFAs [34,35,36,37,38].

However, the existing WHO guidance indicates that total fat should not exceed 30% of total energy intake. WHO recommends that 10% (the upper limit) or less of calories come from saturated fat, and 1% (the upper limit) or less from trans-fats for all individuals, including both adults and children. The recommendations suggest that SFAs and TFAs should be replaced with polyunsaturated fats, and clearly state that there is no reason to increase the intake of SFA if one currently consumes less than 10% of calories from it and no reason to increase TFAs if one currently consumes less than 1% of calories from it.

Improving dietary habits is a societal as well as an individual responsibility. It demands a population-based, multisectoral, and culturally relevant approach. Virtually eliminating TFAs intake and reducing the intake of SFAs is one of the strategic interventions under the area of prevention and reduction of risk factors in the Regional Framework for Action on NCDs [39].

## 2. Objective of the Review and Methodology Used

### 2.1. Objective

The objective of this review is to present an up-to-date overview of the current dietary fat (SFAs and TFAs) reduction initiatives in countries of the EMR by highlighting national and regional programs, strategies and activities aiming at characterizing and/or decreasing the intakes of dietary fat (SFAs and TFAs). The review also aims at:

1. Providing baseline information on dietary intakes and dietary sources of SFAs and TFAs in countries of the Eastern Mediterranean Regional Office (EMRO) region.
2. Providing an overview of national initiatives for the reduction of the intakes of specific types of dietary fat (SFAs and TFAs) in countries of the region.
3. Set up recommendations to accelerate the implementation of WHO’s evidence-based recommendations on SFAs and TFAs intake reduction.

### 2.2. Methods

This paper is a literature review focused on findings retrieved from the online database on fat intake in the EMR. These were accessed in June 2016 and May 2018 using PubMed, WHO and FAO libraries, along with Good Scholar. The terms used to search data were “Fat, SFAs, TFAs” “Pattern” AND/OR “Intake” OR “Consumption” AND “EMRO” OR “EMR” OR the name of the country e.g., “Jordan”. Additional data are retrieved from the WHO Headquarters “e-Library of Evidence for Nutrition Action (eLENA)” and Regional Office of the EMR “Regional Health Observatory”.

## 3. Fat Intake in the EMR

### 3.1. Total Fat (TF) Intake

**Figure 2:**
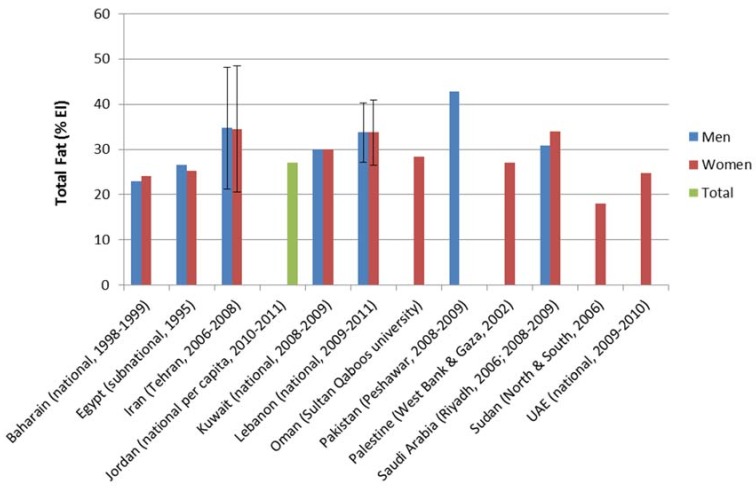
Estimates of total fat intake based on dietary assessment studies in countries of the Eastern Mediterranean Region. EI = energy intake.

The FAO Statistical Databases (FAOSTAT) on food availability in the EMR show a gradual and significant rise in daily fat supply per capita over the past four decades in most countries of the region (FAOSTAT) [40,41]. As shown in Table 2, it is estimated that between 1969 and 2014, the daily average of dietary fat supply in selected countries of the EMR has increased by 26 g (from 52.8 g to 78.8 g/day). Fat supply has, in fact, almost doubled over the past 4 decades in many countries of the region, including Iran, Jordan, Kuwait, Lebanon, Syria and Saudi Arabia. Nearly half of countries of the EMR (Iran, Jordan, Kuwait, Lebanon, Libya, Syria, Tunisia and the United Arab Emirates) have fat supply levels at or above the reported world average (81.8 g/person/day) [40,41]. Food consumption surveys conducted in countries of the region have confirmed the same increasing trend in fat consumption (Figure 2).

### 3.2. TFA and SFA Intake in the EMR

**Figure 3:**
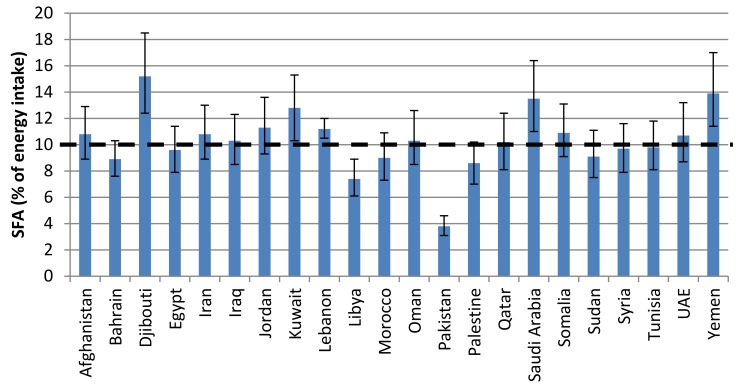
Saturated fat intake in countries of the Eastern Mediterranean region based on a Bayesian model [42].

**Figure 4:**
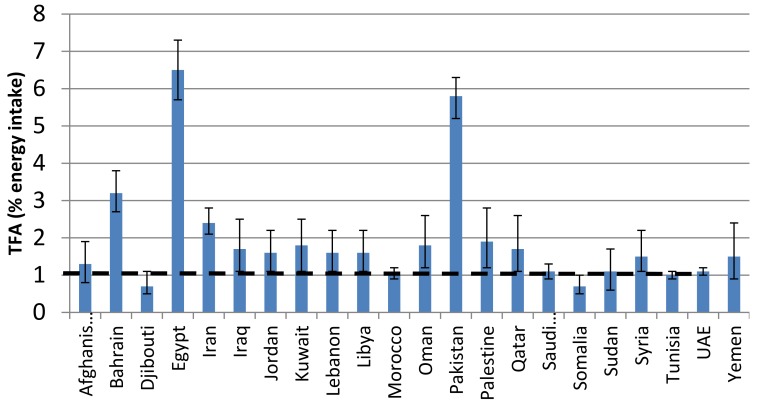
Trans fat intake in countries of the Eastern Mediterranean region based on a Bayesian model [42]. — (dotted line) = WHO Upper Limit.

Based on a multilevel Bayesian hierarchical model, Micha et al. [42] provided estimates for global and regional consumption of dietary fats. According to values reported for countries of the EMR, the average SFAs intake is estimated at 10.3% of energy intake (EI), thus exceeding the global mean consumption level of 9.4% EI (Figure 3). The average TFAs intake in EMR countries is estimated at 1.9% EI, which also exceeds the global average value of 1.4% EI (Figure 4). The North Africa/Middle-East region was reported amongst the regions with the higher levels of TFAs intake. The higher SFAs intake was reported from Djibouti, Kuwait, Saudi-Arabia and Yemen, while the higher TFAs intakes were reported from Egypt and Pakistan.

**Figure 5:**
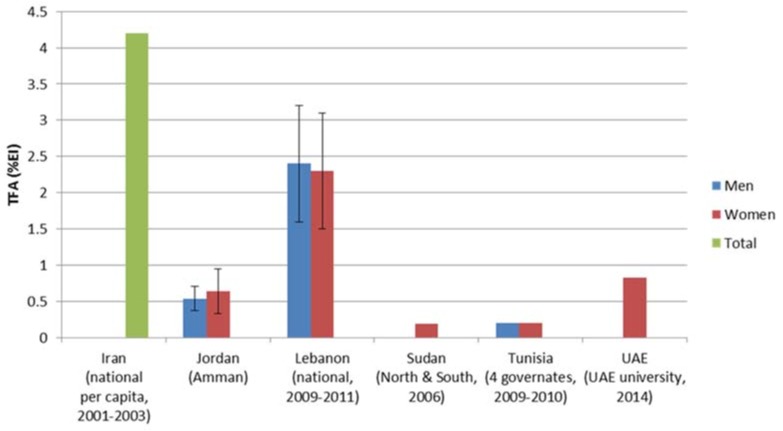
Estimates of TFA (trans fatty acids) intake based on dietary assessment studies in countries of the Eastern Mediterranean Region.

**Figure 6:**
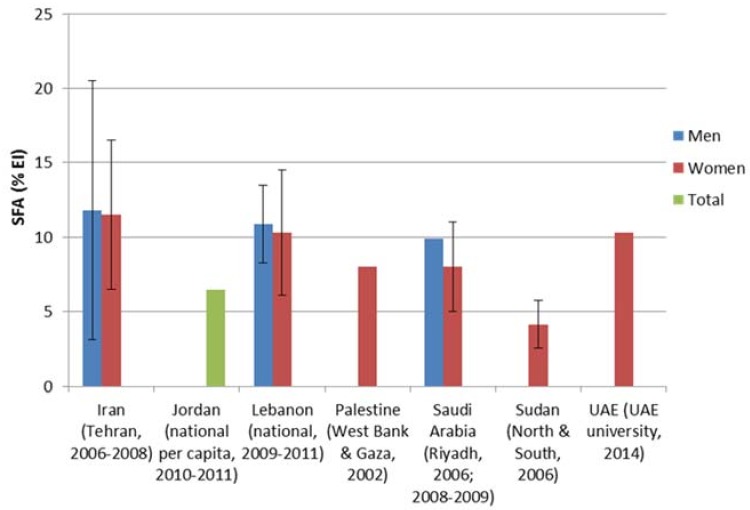
Estimates of SFAs (saturated fatty acids) intake based on dietary assessment studies in countries of the Eastern Mediterranean Region.

Few studies reported on TFAs consumption levels in the EMRO region; and estimates were found to range between 0.1% EI in Tunisia (based on the national survey conducted amongst adults in 2005) and to reach as high as 4.2% EI in Iran (based on per capita household assessment of dietary intake in 2007 (Figure 5). As for SFAs consumption levels, intake estimates amongst adults were found to be relatively high, with most countries exceeding the 10% upper limit. Intake estimates as high as 15% EI were reported from Morocco and Saudi Arabia (Figure 6). It is worthy to note that intra-country discrepancies in the levels of fat, TFAs or SFAs intakes were sometimes observed. This may be explained by differences in study design, targeted age group, dietary assessment method used, and type of food composition database adopted for dietary fat, TFAs, and SFAs intake estimation, amongst other factors.

## 4. Sources of TFAs and SFAs in Commonly Consumed Foods in Countries of the Eastern Mediterranean Region

Literature shows a large variation in TFAs and SFAs content in food, depending on type of food, brand and country. The following food products have been identified as a source of high content of TFAs in the Region:

(1) Margarines and biscuits: Pakistan had the highest TFAs content for both margarine (range: 2.2–34.8% of TF) [43] and biscuits (range: 9.3–34.9% of TF) [44], followed by Iran (margarine: 16.1% of TF; biscuits: range: 23.2–24.5% of TF) [45] and Morocco (margarine (range): 9.1–21.7% of TF) [46]. In Saudi-Arabia, three out of the four analyzed brands of margarine had TFAs content exceeding 2% of total fat (range: 0.2–8.3% of TF) [47], while in Tunisia, one out of the two analyzed margarine brands exceeded 2% (range: 1.4–9.8% of TF) [48]. For biscuits, one third of the samples analyzed in Lebanon exceeded 5% of TF (range: 0.2–19.5% of TF) [49] and one eighth of the analyzed samples in Jordan had TFAs content exceeding 5% of TF (range: 0.7–7.0% of TF) [50]. Elevated TFAs content was recorded in Tunisian classic margarine (9.8% of TF).
(2) French fries: Pakistan had the highest TFAs content in food items such as French fries (range: 0.11–24.00% of TF) [51].
(3) Cereal-based foods: Pakistan had the highest TFAs content in cereal-based foods (range: 2.5–16.3% of TF) [44].
(4) Fast Food, snacks, milk and bakery products: TFA content was high in Iranian food products such as fast food (range: 23.6–30.7% of TF) [52], milk (range: 9.2–14.1% of TF), as well as bakery items (range: 4.5–36.1% of TF) [51]. Lebanon recorded an elevated content of TFAs in bakery products (4.91 ± 3.11%, range: 0.10–6.28% of TF) as well as snacks (8.85 ± 8.57%, range: 0.19–20.85% of TF) [49].
(5) Pie and cake: reported high TFAs in Tunisia: pie (12.7% of TF), and cake (3.1% of TF) [53].

However, TFA content has been reduced over time for edible oils in Iran (range: 0.17–5.40% of TF) as a result of food standards limiting TFA content in edible oils [45].

Countries reporting lower levels of TFAs are e.g., Jordan (snacks: 3.93 ± 8.85%, range: 0.73–41.11%; and bread & bakery: 2.46 ± 0.97%, range: 1.35–4.40% of TF) [50,54] and Morocco, where lower TFAs content was reported for fast foods (average: 1.6 ± 1.1, range: 0.75–2.66% of TF) and traditional foods (average: 2.1 ± 1.9, range: 0.29–6.30% of TF), with TFAs content in most analysed foods being below 5% of total fat [46]. In Tunisia: relatively low TFAs levels were recorded with most food items ranging between 0.7% and 1.4% of total fat [53].

The following food products in the Region have been identified as containing suspected high content of SFA:

(1) **Margarine, mayonnaise and oils**: few standards limiting SFAs in food items were found for the EMR in the literature. The one standard found was an upper limit of 30% for SFAs in edible oils in Iran (passed in November 2007) [45]. SFAs content in solid oils and liquid frying oils are on average 32.07% and 26.77% of total fat, respectively [45], indicating that not all edible oils are within the current standards. Other fat-based food items that have been analyzed for SFA content in Iran include animal butter (67.0%), margarine (42.4%) and mayonnaise (18.1–24.9%) [44,51,54]. Margarine fatty acid composition has also been assessed outside Iran, with margarine in Pakistan having the higher SFAs content (24.2–58.1%) [55,56] and Saudi Arabia having lower SFAs content (19.8–29.3%) [47] compared to Iran [44,54]. Moreover, dairy products, which are also major sources of SFAs in the diet, contain SFAs content of around 50% of total fat in Kuwait [57] and 52.8% to 78.5% of total fat in Iran [57,58]. Vulnerable groups are usually accessible to cheap oils such as Palm oil which have high levels of SFAs.
(2) **Traditional and fast food**: Several studies have been conducted on the fatty acid content of commonly consumed fast foods and traditional foods. With respect to SFAs content in fast foods, Moroccan fast foods had a high contribution of SFAs to total fat (44.3%) [46]. Inversely, significantly lower SFAs were recorded for fast foods in Iran (21.5–38.4%) [52,59] and Bahrain (28.4%) [59,60]. Interestingly, when comparing local to Western fast foods in Bahrain, a similar SFA contribution to total fats was recorded (27.3% and 29.5%, respectively) [60,61]. A similar trend can be seen when comparing Moroccan fast foods to traditional foods where the average SFAs content is 44.3% and 43.1%, respectively. Among Moroccan traditional foods, red meat dishes were relatively high in SFA [46], while Kuwaiti traditional foods had a far lower SFAs contribution to total fat than was seen in Morocco. In Kuwaiti dishes, SFAs content varied per food group with fish dishes having a relatively high SFAs contribution to total fat (29.1%) and vegetable-based dishes had relatively low SFAs (14.6%) [57].

All the priority areas and interventions proposed have a sufficiently strong case—based on research evidence, country experience and expert analysis of the measures—to warrant recommending their adoption

## 5. International Experience on Reducing TFA and SFA Intake and Lessons Learned

Experience from countries proved that the reduction of SFAs and TFAs is possible and has major impact on public health. The reduction of the dietary intake of SFAs has been remarkably successful in bringing down deaths from coronary heart disease and strokes [62]. CHD mortality has declined in Finland (North Karelia project) by 55% among men and 68% among women between 1972 and 1992. Dietary surveys were carried out in connection with these surveys in 1982 and 1992. The total fat content of the Finnish diet changed from 38% of energy to 34%, saturated fat decreased from 21 to 16%, and polyunsaturated fat from 3 to 5% and the intake of cholesterol decreased by 16%. Dietary changes seem to explain the decrease in serum cholesterol. Together with a decline in smoking among males, as well as better blood pressure control, they have contributed to the dramatic decline in CHD mortality in Finland [63].

Globally, increased intake of TFAs is estimated to be responsible for more than 500,000 deaths per year. An overview of national policies has concluded that the most effective way of ensuring a significant fall in TFAs intakes is by legally prohibiting the sale of food products containing industrially produced TFAs. In practice, highly effective legislation (such as that in Austria, Denmark, Sweden, Norway, Hungary, Iceland and Switzerland) indicates a limit of 2 g/100 g of oils or fats, and soft spreadable margarines, and no more than 5% for all other foods [64]. The voluntary reduction approach taken by some countries requires a solid and sustainable monitoring system and has not been proven to be as effective [64]. In 2005, Canada became the first country to implement mandatory labelling of TFAs. In 2006, a Canadian Trans Fat Task Force recommended that TFAs should not exceed 2% of total fat content for vegetable oils and soft spreadable margarines, and no more than 5% for all other foods. In the United States, a cost-benefit analysis comparing the health benefits of TFAs reduction with the expense of labelling led to the mandated inclusion of TFAs content on food labels which increased awareness of consumers to select the food with low levels of TFAs [65].

The Danish Nutrition Council reported in 2001 that about 50,000 Danes were at high risk for CVD as a direct result of their intake of TFA. Legislation to limit the content of trans fat in Danish food was presented to Parliament in 2003 and approved. Studies on the efficacy of this legislation illustrate that artificial TFAs are now “virtually eliminated” from Danish food[65]. The data show that the decline in CHD mortality rates in Denmark for the period 1980–2009 was the largest in the EU (70%) [65].

## 6. Regional Strategies to Reduce Fat (Total Fat, SFAs & TFAs) Intake at Population Levels

Policies aimed at restricting the TFAs and SFAs content of food were associated with significant reductions in TFAs and SFAs levels and replacing them with PUFAs, such as olive oil, cold-water fish (salmon, tuna, sardines, cod and anchovy), vegetable oils, flax seeds, walnuts and some types of vegetables. Such policies are feasible, achievable and likely to have an effect on public health [5,66,67]. The Regional policies are guided by the WHO World Health Assembly (WHA) and WHO Regional Committee (RC) resolutions which are aiming to:

(1) **Reduce premature mortality from NCDs by 25%**: The Political Declaration of the United Nations General Assembly on the Prevention and Control of Non-Communicable Diseases in September 2011 [68] prompted the WHO Regional Office for the Eastern Mediterranean to spearhead a salt and fat reduction initiative in the region. In May 2013, the World Health Assembly endorsed the WHO Global Action Plan for the Prevention and Control of NCDs 2013–2020. This Global Action Plan provides Member States, international partners and WHO with a road map and menu of policy options based on nine global NCDs targets, to be attained by 2025, including the number one target: to achieve a 25% relative reduction in premature mortality from NCDs by 2025. The 59th session of the WHO Regional Committee for the Eastern Mediterranean (2012) adopted the resolution EM/RC59/R.2, thus endorsing the regional Framework for Action on the commitments of Member States to implement the United Nations Political Declaration on Non-Communicable Diseases [1]. In its EM/RC59/R.2 resolution, the WHO EMRO urged the Member States to implement the core set of interventions in the regional Framework for Action, with these interventions including the reduction of the population’s salt intake levels and the replacement of trans fat with polyunsaturated fat [69,70,71].
(2) **Halt diabetes and obesity**: WHO EMRO is working closely with governments to achieve the seventh global target of the Global strategy on diet, physical activity and health that aims to halt the rise in diabetes and obesity [5]. “Proposed policy priorities for preventing obesity and diabetes in the Eastern Mediterranean Region also published in 2017” is a recent publication by WHO EMRO which includes a set of evidence-based population-level recommendation for Member States to implement in order to prevent obesity and diabetes [71]. A policy statement and action plan on the reduction of fat intake and the lowering of heart attack rates in the Eastern Mediterranean region was also issued on 2013 by WHO EMRO [72]. The policy goals are to:

- eliminate all industrially produced trans fats from the food supply; and
- reduce markedly the saturated fat content of the food supply.

According to the data contained in the WHO Global database on the implementation of nutrition action (GINA), recently updated with information from the second global nutrition policy review (2016). A significantly higher number of countries [72] have national policies and plans that contain explicit goals and strategies to improve nutrition and promote healthy diets (2017), the number of countries that are taking action has increased in the following areas: food reformulation, from 29 in 2009–2010 to 60 in 2016–2017 (in 40 countries the focus is on sodium/salt reduction); TFAs bans, from 12 to 26; and fiscal policies to promote healthy diets, from 15 to 38 [5], only few countries from the Region.

## 7. Examples of Action Taken by Countries of EMR

In the EMR, several countries have initiated fat reduction initiatives. However, in most countries of the region, advocacy groups or research institutions are undertaking these initiatives in the absence of policies.

The following are feasible actions taken by Member States which will have an impact at population level:

(1) **The Gulf Cooperation Council (GCC)**: Standardization Organization: The Gulf Standardization office (GSO) provides standards for food policy in 7 Member States (Bahrain, Kuwait, Oman, Qatar, Saudi Arabia, United Arab Emirates and Yemen). This includes mandatory nutritional labelling of fat (total fat, TFAs, SFAs, PUFA, MUFA) as g/100 g and % daily value (DV) [73]. Progress is also being made towards the reduction of dietary TFAs through a project by the GSO (2013) which aims at limiting the maximum level of TFA for hydrogenated oils and spreadable vegetarian margarine to 2% of total fat and the maximum level for all other foods containing TFA to 5% of total fat [5]. Enforced implementation is still a challenge and not effective yet.
(2) **Iran**: executive committee, composed of members from the Ministry of Health and Medical Education, Ministry of Industry, Ministry of Agriculture, Ministry of Commerce and the National Standard Organization, was established in 2004 to develop an operational plan for reducing SFAs and TFAs in edible oils in Iran. In 2005, the Ministry of Commerce was obliged to gradually replace the hydrogenated oils as the subsidized ones, by non-hydrogenated (especially olive oil) and liquid frying oils [74]. In 2008, the Ministry of Health and Medical Education and National Standard Organization were obliged to revise the instructions of packaging and mandate manufacturers and importers to affix labels to all food products, especially edible oils. Also, in 2008, the National Standard Organization was mandated to revise standard NO.9131, so that SFAs and TFAs contents of edible oils (both imported and locally produced ones) are limited to 25% and 5%, respectively. As of 2011, Ministry of Health and Medical Education, Industry, Agriculture, Commerce and National Standard Organization developed a national policy for edible oil safety. In 2014, the High Council of Health and Food Security approved to revise the standards of TFAs to less than 2% and saturated fatty acid to less than 25%. In order to reduce saturated fatty acid, the Ministry of Trade was asked to reduce the amount of palm oil import, so in 2014, palm oil import was reduced from 70% to 30%. As a result of these legislations, both palm oil imports and TFAs content in edible oil has been significantly reduced (information provided by nutrition focal point) [74,75].
(3) **Iraq**: Subsidy on palm oil and hydrogenated ghee removed and replaced by other types of oil.
(4) **Jordan**: banning the addition of vegetable oils to dairy products including palm oil through national food standards.
(5) **Tunisia**: In 2015, one manufacturer has just launched a kind of margarine without trans fat after adapting new food processing technology.
(6) **Morocco**: a draft resolution prepared and submitted to the parliament. However, the advocacy group from Ministry of Health (MOH) and academia are active and contributed to increasing the awareness of the population, they have succeeded in bringing attention of the industry to cut fat on a voluntary basis in dairy products, but this is still premature.

Many countries are relying on health education and awareness campaigns and initiation of community-based communications strategies to the public such as Egypt, UAE, Bahrain, Pakistan, Palestine, Lebanon, Kuwait and Qatar.

## 8. Data Quality and Availability

Accuracy and availability of data is a key challenge in the region**, s**everal Member States have attempted to evaluate SFAs and TFAs intake levels based on dietary assessment approaches, including food frequency questionnaires, dietary records or 24-h dietary recalls. One of the biggest challenges for the assessment of SFAs and TFAs intakes based on dietary assessment tools is the availability of up-to-date, culture-specific food composition tables. However, several Member States recurred to the use of food composition databases published by Western developed countries such as the US, the UK or France. Support research in this area of work is a priority to understand the food consumption pattern in the Region and monitor the implementation of the policies and assess their impacts.

## 9. Discussion

The Region is still struggling with the implementation of WHO recommendations to reduce TF, SFAs and TFAs due to the weaknesses of health and trade policies and commitments in most of the countries. Low- and middle-income countries facing political and economic problems delays the implementation of such interventions and all focus goes towards curative medical services [5]. However, during the last five years, scaling up of NCD prevention policies is being highlighted at higher political levels with the constant support from WHO and civil societies. Removing subsidies on oil or shifting it to cash transfer or other healthy food items is working in many countries such as Egypt, Jordan and GCC. Countries like Pakistan and Egypt where TFA intake is the highest should take immediate actions. The same applies for Djibouti, Kuwait, Saudi-Arabia and Yemen to reduce SFAs intake [68].

Reducing SFAs and TFAs policy is unique in its combination of efficacy; cost-effectiveness with high impact on the public, through adopting policies limiting SFAs/TFAs which content in food would reduce the national implementation and monitoring costs. Legally limiting trans-fat content in food would have no major negative consequences for both the industry and consumers, and doing so may contribute to reducing inequalities. Removing SFAs and TFAs from the food supply is possible and straightforward public health intervention for reducing CVD risk and improving nutritional quality of diets. Local oil refining companies would easily eliminate the production of TFAs through introducing mandatory regulations. However, it is important in a region where food imports often comprise a substantial proportion of the national food supply to set food standards and specification to decrease the content of SFAs and TFAs in imported foods and oils and increase PUFAs similar to the experience of Singapore [5,76].

The high burden and increasing secular trend of obesity and NCDs in countries of the EMR require immediate public health attention. The toll of NCD-related morbidity and mortality can be considerably decreased if evidence-based preventive interventions are implemented effectively [2,5]. In this context, within the recommended total fat intake, more focus should be given to the quality and the type of oil used or subsidized, to protect vulnerable groups including the poor from consuming cheap oils and margarines made of kernel palm oil or palm oil. Reductions in SFAs and TFAs intakes have been highlighted as cost-effective strategies that may hamper the growth of the NCDs epidemic [5,77]. This review emphasizes the high intake levels of total fat, TFAs and SFAs in countries of the EMRO region, and highlights the need for more data on TFAs intake levels. There is a wide spectrum in total fat, TFAs and SFAs reduction initiatives that are currently being undertaken in countries of the region [2,78,79].

Implementation of appropriate restrictions on marketing of unhealthy food to children: including diet high in TFAs, SFAs, salt and sugar is a top priority in the Region. The Regional office developed the tools to help countries including the Nutrients Profiling Model. Nutrient profiling is “the science of classifying or ranking food according to its nutritional composition for reasons related to preventing disease and promoting health” [80]. Adopting the nutrient profiling developed by the WHO Regional Office has at least two important advantages. Firstly, it will limit the diversity of the criteria applied across the Region to determine which food is healthy and which is not. It will therefore facilitate comparisons as well as the development of common of cross-border standards on the marketing of unhealthy food to children. Secondly, this model offers a clear, simple method to distinguish healthy and unhealthy food, and is built on objective, evidence-based analyses [80]. Therefore, its adoption is likely to protect countries from possible challenges from the food industry based on international trade rules, and in particular the principle of non-discrimination which is at the heart of the law of the World Trade Organization [80].

## 10. Conclusions and Recommendations

In conclusion, a legal limit for food produced locally or imported appears as the option with the most potential to reduce the availability and consumption of trans fats and saturated fatty acids, as voluntary reformulation might not work in some settings and, for some countries, knowing that some countries import more than 70% of their food. Other advantages of a policy limiting TFAs and SFAs content in food include low implementation and monitoring costs, as well as low cost to industry, and doing so may contribute to reducing inequalities. Removing TFAs from the food supply is possibly one of the most straightforward public health interventions to reduce CVD risk and improve nutritional quality of diets.

The following are representing the strategic and cost-effective intervention set by WHO EMR to reduce SFAs and TFAs intake at population levels, which has an impact across the life-course [5]. These recommendations are in line with the new WHO initiative “Replace” to eliminate TFAs in foods by 2030 [79]:

(1) **Strengthening of political commitment**: countries of the region are encouraged to strengthen the political commitment to the reduction of TFAs and SFAs intakes as one of the most cost-effective strategies to hamper the growth of obesity and NCDs that are plaguing the economies of countries of the region. This can be achieved by organizing politician briefings as well as regular one-to-one meetings with relevant governmental officials.
(2) **Fiscal measures**: progressively eliminate national subsidies for all types of fats/oils and introduce an effective tax on high-fat and/or high-sugar foods.
(3) **Publicly funded food**: procurement and provision of healthy food in public institutions, such as government canteens, hospitals, universities, schools and kindergartens through setting mandatory nutrition standards. All countries are recommended to:

- Implement mandatory nutrition standards across all public institutions, through (a) application of the Regional nutrient profile model (b) introduction of meal standards, and (c) measures to eliminate the sale of foods or drinks high in fat, sugar or salt.
- Issue mandatory guidelines for the revision of procurement to provide healthy food, including limiting the volume of fats/oils in public-sector catering facilities in order to facilitate the necessary and properly documented menu changes.
- Provide guidance and training on appropriate catering methods to limit the use of frying foods and help design menu changes.

(4) **Food supply and trade**: regulate all food produced locally or imported by setting benchmarks on the recommended levels of TFAs and SFAs, as well as limiting the imports of palm oil or using it in the food industry or processing. Marketing: Implement the WHO Set of Recommendations on Marketing of Foods and Non-alcoholic Beverages to Children and consider mandatory restrictions to eliminate all forms of marketing of foods high in fat, sugar and salt to children and adolescents (up to age 18) across all media, according to the Regional Action Plan to Address Unopposed Marketing of Unhealthy Food and Beverages.
(5) **Support research for assessing SFAs/TFAs intake and contents in foods**: It is recommended that data from the region be enhanced by additional investigations conducted in individual Member States, particularly in countries where a lack of data is still noticeable.
(6) **Implementation of appropriate restrictions on marketing of unhealthy food to children**: including diet high in TFA and SFA, low in salt and sugar.
(7) **Standardization of regional food composition tables**: it is recommended to mark standardized Food Composition Tables with more focus on traditional diets and reflecting the content of TFAs and SFAs in the foods through expanding the regional initiative led by WHO, and other International organization.
(8) **Product Reformulation**: Member States should strive to collaborate with food producers (industry, catering companies, restaurants) for the reformulation of processed and catered foods with the aim of decreasing total fat, TFAs and SFAs content of processed foods.
(9) **Food Labelling**: implement a mandatory front-of-pack labelling scheme with elements to enable consumers to interpret information easily (such as colour coding or the use of terms such as “high”, “medium”, “low”).
(10) **Raising consumer awareness**: a continuum of activities aiming to raise fat-related consumer awareness should be planned at the national level rather than engaging in sporadic and intermittent awareness activities. Success in raising consumer awareness may require a partnership between Non-Government Organization (NGOs), industry, media, the health sector and national platforms. Member states are encouraged to participate and develop campaigns with clear objectives and messages, and to develop campaign-related materials such as educational pamphlets, posters and websites.
(11) **Social support**: Review government safety-net and social support policies to include healthy foods (e.g., subsidies for the poor allowing purchase of foods with only modest amounts of total fat and low saturated fat content).
(12) **Monitoring and evaluation**: Those countries that have baseline data on actual TFAs and SFAs intakes and their levels in foods, and that have launched fat reduction initiatives are encouraged to embrace monitoring approaches.

## Figures and Tables

**Table 1:** Estimated deaths (1000) by cardiovascular diseases in the Eastern Mediterranean Region, 2016 (World Health Organization, 2018).

Country	Total Deaths by CVDs	Total Deaths in EMR	% of Deaths Due to CVDs
Afghanistan	51.2	248.2	20.6
Bahrain	0.8	2.8	27.8
Djibouti	1.4	7.4	18.8
Egypt	245.9	608.4	40.4
Iran	160.8	371.5	43.3
Iraq	51.6	189.6	27.2
Jordan	13.4	36.4	36.7
Kuwait	4.6	11.0	41.3
Lebanon	17.8	38.3	46.5
Libya	11.6	33.7	34.6
Morocco	69.5	182.0	38.2
Oman	4.0	11.2	36.0
Pakistan	411.6	1403.1	29.3
Qatar	1.1	4.0	26.6
Saudi Arabia	42.4	113.5	37.4
Somalia	16.0	167.0	9.6
Sudan	80.3	281.9	28.5
Syrian	37.9	150.4	25.2
Tunisia	32.0	72.1	44.3
UAE	6.0	15.1	39.5
Yemen	56.8	174.1	32.6
Regional	1316.6	4121.9	31.9

CVDs = cardiovascular diseases; EMR = Eastern Mediterranean Region.

**Table 2:** Changes in dietary fat supply (g/person/day) from 1969–1971 to 2002–2004 in selected countries of the Eastern Mediterranean region (FAOSTAT—The Food and Agriculture Organization Corporate Statistical Database).

Fat Supply (g/day)	1969–1971	1979–1981	1995–1997	2001–2003	2002–2004	2005	2006	2007	2008	2009	2010	2011	2014
Djibouti	34	36	54	65	57	66	65	68	69	63	56	60	60
Egypt	47	65	57	58	56	56	57	62	62	60	62	64	57
Iran	39	60	66	62	63	63	68	73	74	77	76	74	76
Jordan	52	62	76	80	74	90	94	95	87	92	98	101	94
KSA	33	76	73	82	78	84	96	81	82	82	92	96	82
Kuwait	69	88	98	113	102	116	124	123	126	122	122	116	123
Lebanon	63	82	103	113	103	117	107	110	107	109	108	106	108
Libya	62	91	102	94	93	97	93	95	96	94	95	95	-
Morocco	43	52	60	59	54	57	62	65	64	65	64	65	61
Palestine	-	-	67	63	69	62	53	55	51	52	50	48	-
Sudan	65	74	65	74	68	66	-	-	-	-	-	-	-
Syria	60	83	99	101	91	104	107	96	99	104	104	107	-
Tunisia	63	70	86	94	83	90	92	85	95	87	86	87	87
UAE	97	130	107	92	92	74	82	84	90	92	91	103	83
Yemen	29	38	34	41	44	47	49	48	45	44	43	45	47

KSA = Kingdom of Saudi Arabia; UAE = United Arab Emirates.

## References

[1] World Health Organization (2014). Global Status Report on Noncommunicable Diseases 2014.

[2] World Health Organization, Regional Office for the Eastern Mediterranean (EMRO) (2017). Eastern Mediterranean Region Framework for Health Information Systems and Core Indicators for Monitoring Health Situation and Health System Performance. http://applications.emro.who.int/docs/EMROPUB_2017_EN_16766.pdf?ua=1.

[3] GBD 2016 Causes of Death Collaborators (2017). Global, regional, and national age-sex specific mortality for 264 causes of death, 1980–2016: A systematic analysis for the Global Burden of Disease Study 2016. Lancet.

[4] World Health Organization Regional, Office for the Eastern Mediterranean (EMRO) (2012). Health Systems Strengthening in Countries of the Eastern Mediterranean Region: Challenges, Priorities and Options for Future Action.

[5] Alwan A., McColl K., James J.P., Al-Jawaldeh A. (2017). Proposed Policy Priorities for Preventing Obesity and Diabetes in the Eastern Mediterranean Region.

[6] World Health Organization Global Observatory (GHO) Data. http://www.who.int/gho/en/.

[7] World Health Organization, Global Health Observatory Data Repository Raised Total Cholesterol (≥5.0 mmol/L): Data by Country. http://apps.who.int/gho/data/view.main.2467.

[8] Lichtenstein A.H. (2014). Dietary trans fatty acids and cardiovascular disease risk: Past and present. Curr. Atheroscler. Rep..

[9] Mozaffarian D., Clarke R. (2009). Quantitative effects on cardiovascular risk factors and coronary heart disease risk of replacing partially hydrogenated vegetable oils with other fats and oils. Eur. J. Clin. Nutr..

[10] Sun Q., Ma J., Campos H., Hankinson S.E., Manson J.E., Stampfer M.J., Rexrode K.M., Willett W.C., Hu F.B. (2007). A prospective study of trans fatty acids in erythrocytes and risk of coronary heart disease. Circulation.

[11] World Health Organization (2003). Diet, Nutrition and the Prevention of Chronic Diseases: Report of a Joint WHO/FAO Expert Consultation.

[12] Wang Q., Afshin A., Yakoob M.Y., Singh G.M., Rehm C.D., Khatibzadeh S. (2016). Impact of nonoptimal intakes of saturated, polyunsaturated, and trans fat on global burdens of coronary heart disease. J. Am. Heart Assoc..

[13] Welty F.K. (2013). How do elevated triglycerides and low HDL-cholesterol affect inflammation and atherothrombosis?. Curr. Cardiol. Rep..

[14] Bhardwaj S., Passi S.J., Misra A. (2011). Overview of trans fatty acids: Biochemistry and health effects. Diabetes Metab. Syndr..

[15] Farvid M.S., Ding M., Pan A., Sun Q., Chiuve S.E., Steffen L.M., Willett W.C., Hu F.B. (2014). Dietary linoleic acid and risk of coronary heart disease: A systematic review and meta-analysis of prospective cohort studies. Circulation.

[16] Melanson E.L., Astrup A., Donahoo W.T. (2009). The relationship between dietary fat and fatty acid intake and body weight, diabetes, and the metabolic syndrome. Ann. Nutr. Metab..

[17] Micha R., Mozaffarian D. (2010). Saturated fat and cardiometabolic risk factors, coronary heart disease, stroke, and diabetes: A fresh look at the evidence. Lipids.

[18] Chowdhury R., Warnakula S., Kunutsor S., Crowe F., Ward H.A., Johnson L., Franco O.H., Butterworth A.S., Forouhi N.G., Thompson S.G. (2014). Association of dietary, circulating, and supplement fatty acids with coronary risk: A systematic review and meta-analysis. Ann. Intern. Med..

[19] Dashti B., Al-Awadi F., Sawaya W., Al-Otaibi J., Al-Sayegh A. (2003). Fatty acid profile and cholesterol content of 32 selected dishes in the state of Kuwait. Food Chem..

[20] Anzid K., Baali A., Vimard P., Levy-Desroches S., Cherkaoui M., López P.M. (2014). Inadequacy of vitamins and minerals among high-school pupils in Ouarzazate, Morocco. Public Health Nutr..

[21] Smit L.A., Mozaffarian D., Willett W. (2009). Review of fat and fatty acid requirements and criteria for developing dietary guidelines. Ann. Nutr. Metab..

[22] World Health Organization (2009). Global Health Risks: Mortality and Burden of Disease Attributable to Selected Major Risks.

[23] Jakicic J.M., Ard J.D., de Jesus J.M., Houston Miller N., Hubbard V.S., Lee I.M., Lichtenstein A.H., Loria C.M., Millen B.E., Nonas C.A. (2013). AHA/ACC Guideline on Lifestyle Management to Reduce Cardiovascular Risk. Circulation.

[24] Mozaffarian D., Katan M.B., Ascherio A., Stampfer M.J., Willett W.C. (2006). Trans fatty acids and cardiovascular disease. N. Engl. J. Med..

[25] Stachowska E., Dołegowska B., Olszewska M., Gutowska I., Chlubek D. (2004). Isomers of trans fatty acids modify the activity of platelet 12-P lipoxygenase and cyclooxygenase/thromboxane synthase. Nutrition.

[26] Bray G.A., Lovejoy J.C., Smith S.R., DeLany J.P., Lefevre M., Hwang D., Ryan D.H., York D.A. (2002). The influence of different fats and fatty acids on obesity, insulin resistance and inflammation. J. Nutr..

[27] Risérus U., Willett W.C., Hu F.B. (2009). Dietary fats and prevention of type 2 diabetes. Prog. Lipid Res..

[28] Salmeron J., Hu F.B., Manson J.E., Stampfer M.J., Colditz G.A., Rimm E.B., Willett W.C. (2001). Dietary fat intake and risk of type 2 diabetes in women. Am. J. Clin. Nutr..

[29] Kavanagh K., Jones K.L., Sawyer J., Kelley K., Carr J.J., Wagner J.D., Rudel L.L. (2007). Trans fat diet induces abdominal obesity and changes in insulin sensitivity in monkeys. Obesity.

[30] Simopoulos A.P. (2008). The importance of the omega-6/omega-3 fatty acid ratio in cardiovascular disease and other chronic diseases. Exp. Biol. Med..

[31] Innis S.M. (2006). Trans fatty intakes during pregnancy, infancy and early childhood. Atheroscler. Suppl..

[32] FAO/WHO (2010). Interim Summary of Conclusions and Dietary Recommendations on Total Fat and Fatty Acids. Joint FAO/WHO Expert Consultation on Fats and Fatty Acids in Human Nutrition..

[33] Siri-Tarino P.W., Sun Q., Hu F.B., Krauss R.M. (2010). Meta-analysis of prospective cohort studies evaluating the association of saturated fat with cardiovascular disease. Am. J. Clin. Nutr..

[34] WHO Regional Office for Europe Nutrient Profile Model (2015). Copenhagen: World Health Organization Regional Office for Europe. http://www.euro.who.int/__data/assets/pdf_file/0005/270716/Nutrient-children_web-new.pdf.

[35] Ascherio A., Hennekens C.H., Buring J.E., Master C., Stampfer M.J., Willett W.C. (1994). Trans-fatty acids intake and risk of myocardial infarction. Circulation.

[36] Willett W.C., Stampfer M.J., Manson J.E., Colditz G.A., Speizer F.E., Rosner B.A., Sampson L.A., Hennekens C.H. (1993). Intake of trans fatty acids and risk of coronary heart disease among women. Lancet.

[37] Kandhro A., Sherazi S.T., Mahesar S.A., Bhanger M.I., Talpur M.Y., Rauf A. (2008). GC-MS quantification of fatty acid profile including trans FA in the locally manufactured margarines of Pakistan. Food Chem..

[38] World Health Organization The Political Declaration of the United Nations General Assembly on the Prevention and Control of Non-Communicable Diseases: Commitments of Member States and the Way Forward. 2012 EM/RC59/R.2..

[39] World Health Organization Implementing the United Nations Political Declaration on Prevention and Control of Noncommunicable Diseases Based on the Regional Framework for Action. Regional Committee for the Eastern Mediterranean Sixtieth Session Provisional Agenda Item 7. EM/RC60/9 Rev.1 September 2013..

[40] Food and Agriculture Organization (FAOSTAT) (2014). Supply Utilization Accounts and Food Balances Domain: Food Balance Sheets. http://faostat3.fao.org/download/FB/FBS/E.

[41] Food and Agriculture Organization of the United Nations (2014). Food and Nutrition in Numbers 2014. http://coin.fao.org/coin-static/cms/media/22/14163487981020/food_and_nutrition_in_numbers.pdf.

[42] Micha R., Khatibzadeh S., Shi P. (2014). Global, regional, and national consumption levels of dietary fats and oils in 1990 and 2010: A systematic analysis including 266 country-specific nutrition surveys. BMJ.

[43] Kandhro A., Sherazi S.T., Mahesar S.A., Bhanger M.I., Talpur M.Y., Arain S. (2008). Monitoring of fat content, free fatty acid and fatty acid profile including trans fat in Pakistani biscuits. J. Am. Oil Chem. Soc..

[44] Mahesar S., Kandhro A.A. (2010). Determination of total trans fat content in Pakistani cereal-based foods by SB-HATR FT-IR spectroscopy coupled with partial least square regression. Food Chem..

[45] Hajimahmoodi M., Arami S., Nosrati M. (2013). Trans Fatty Acid Content of Iranian Edible Oils. Food Nutr. Sci..

[46] Ministry of Health (2015). Unpublished Report on the Fat Profile for Food Products.

[47] Bakeet Z.A.N., Alobeidallah F.M., Arzoo S. (2013). Fatty acid composition with special emphasis on unsaturated trans fatty acid content in margarines and shortenings marketed in Saudi Arabia. Int. J. Biosci..

[48] National Nutrition Institutes (2015). Unpublished Report Data on Food Consumption Pattern.

[49] Nasreddine L., Naja F.A., Sibai A.M., Helou K., Adra N., Hwalla N. (2013). Trends in nutritional intakes and nutrition-related cardiovascular disease risk factors in Lebanon: The need for immediate action. Leban. Med. J..

[50] Mashal R. (2011). Variability in trans fatty acid content of selected local and imported foods in Jordan. Riv. Ital. Delle Sostanze Grasse.

[51] Karim Z., Khan K.M., Ahmed S., Karim A. (2014). Assessment of Trans Fatty Acid Level in French Fries from Various Fast Food Outlets in Karachi, Pakistan. J. Am. Oil Chem. Soc..

[52] Nazari B., Asgary S., Azadbakht L. (2012). Fatty acid analysis of Iranian junk food, dairy, and bakery products: Special attention to trans-fats. J. Res. Med. Sci..

[53] National Institute of Nutrition Food and Technology Food (1994). Tunisian Food Composition Table of the Tunisian.

[54] Takruri H.R., Alkurd R.A. (2014). Intakes of Fats, Cholesterol, Fiber and Micronutrients as Risk Factors for Cardiovascular Disease in Jordan. Jordan J. Biol. Sci..

[55] Nazari B., Asgary S., Sarrafzadegan N. (2010). Warning about Fatty Acid Compositions in Some Iranian Mayonnaise Salad Dressings. Int. J. Prev. Med..

[56] Rifat-uz-Zaman Z.I., Ali U. (2013). Dietary Intakes of Urban Adolescents of Sialkot, Pakistan Do Not Meet the Standards of Adequacy. Pak. J. Nutr..

[57] Zaghloul S., Al-Hooti S.N., Al-Hamad N., Al-Zenki S., Alomirah H., Alayan I., Al-Attar H., Al-Othman A., Al-Shami E., Al-Somaie M. (2013). Evidence for nutrition transition in Kuwait: Over-consumption of macronutrients and obesity. Public Health Nutr..

[58] Bahadoran Z., Mirmiran P., Golzarand M., Hosseini-Esfahani F., Azizi F. (2012). Fast food consumption in Iranian adults; dietary intake and cardiovascular risk factors: Tehran Lipid and Glucose Study. Arch. Iran. Med..

[59] Asgary S., Nazari B., Sarrafzadegan N., Parkhideh S., Saberi S., Esmaillzadeh A., Azadbakht L. (2009). Evaluation of fatty acid content of some Iranian fast foods with emphasis on trans fatty acids. Asia Pac. J. Clin. Nutr..

[60] Gharib N., Rasheed P. (2011). Energy and macronutrient intake and dietary pattern among school children in Bahrain: A cross-sectional study. Nutr. J..

[61] Gharib N.M., Rasheed P. (2013). Obesity among Bahraini Children and Adolescents: Prevalence and Associated Factors. J. Bahrain Med. Soc..

[62] Oomen C.M., Ocké M.C., Feskens E.J., van Erp-Baart M.A., Kok F.J., Kromhout D. (2001). Association between trans fatty acid intake and 10-year risk of coronary heart disease in the Zutphen Elderly Study: A prospective population-based study. Lancet.

[63] Pirjo P., Vartiainen E., Seppänen R., Aro A., Puska P. (1996). Changes in Diet in Finland from 1972 to 1992: Impact on Coronary Heart Disease Risk. Prev. Med..

[64] World Health Organization (2015). WHO Regional Office for Europe. Eliminating Trans Fats in Europe: A Policy Brief..

[65] Danish Academy of Technical Sciences, ATV Economic Nutrition Policy Tools—Useful in the Challenge to Combat Obesity and Poor Nutrition? December 2007. https://atv.dk/sites/atv.dk/files/media/document/Rapport_ATV_Economic_nutrition_policy_tools_December_2007.pdf.

[66] Jakobsen M.U., O’Reilly E.J., Heitmann B.L., Pereira M.A., Balter K., Fraser G.E. (2009). Major types of dietary fat and risk of coronary heart disease: A pooled analysis of 11 cohort studies. Am. J. Clin. Nutr..

[67] Tutino V., Caruso M.G., De Leonardis G., De Nunzio V., Notarnicola M. (2017). Tissue Fatty Acid Profile is Differently Modulated from Olive Oil and Omega-3 Polyunsaturated Fatty Acids in ApcMin/+ Mice. Endocr. Metab. Immune Disord.-Drug Targets.

[68] General Assembly of the United Nations (2011). Political Declaration of the High-Level Meeting of the General Assembly on the Prevention and Control of Non-Communicable Diseases.

[69] Krenosky S., Mary L., Nora L., Lynne U., Michel V. (2012). Risk Assessment of Exposure to Trans Fat in Canada. Int. Food Risk Anal. J..

[70] World Health Organization (2010). A Review of Nutrition Policies: Draft Report.

[71] WHO Regional Office for the Eastern Mediterranean (EMRO) (2016). Assessing National Capacity for the Prevention and Control of Noncommunicable Diseases.

[72] World Health Organization (2015). Report on the Technical Consultation on Salt and Fat Reduction Strategies in the Eastern Mediterranean Region.

[73] GCC Standardization Organization (2015). Trans Fatty Acids GSO 2483. https://www.gso.org.sa/store/gso/standards/GSO:693058/GSO%202483:2015?lang=en.

[74] WHO (2015). Moving Forward on Salt and Fat Reduction in the Region. East. Mediterr. Health J..

[75] World Health Organization (2014). Policy Statement and Recommended Actions for Reducing Fat Intake and Lowering Heart Attack Rates in the Eastern Mediterranean Region.

[76] World Health Organization (2012). Global Database on the Implementation of Nutrition Action (GINA).

[77] Peymani P., Joulaei H., Lankarani K.B. (2012). Iran’s Experience on Reduction of Trans-Fatty Acid Content in Edible Oils. Middle-East J. Sci. Res..

[78] GCC Standardization Organization The Technical Regulation GSO 9/2013 “Labeling of Prepackaged Foodstuffs”. https://www.gso.org.sa/store/gso/standards/GSO:615544/GSO%209:2013?lang=en.

[79] World Health Organization (2018). An Action Package to Eliminate Industrially-Produced Trans Fat from the Global Food Supply.

[80] Rayner M., Scarborough P., Kaur A. (2013). Nutrient profiling and the regulation of marketing to children. Possibilities and pitfalls. Appetite.

